# Development and Evaluation of Hepatoprotective Polyherbal Formulation Containing Some Indigenous Medicinal Plants

**DOI:** 10.4103/0250-474X.41474

**Published:** 2008

**Authors:** P. M. Dandagi, M. B. Patil, V. S. Mastiholimath, A. P. Gadad, R. H. Dhumansure

**Affiliations:** Department of Pharmaceutics, K. L. E. S's College of Pharmacy, Belgaum-590 010, India; 1 Department of Pharmacognosy and Phytochemistry, K. L. E. S's College of Pharmacy, Belgaum-590 010, India

**Keywords:** Hepatoprotective, polyherbal, *M. charantia*, *N. jatamansi*, *F. asafoetida*

## Abstract

The present study explores the hepatoprotective activity of various extracts of *Ferula asafoetida*, *Momordica charantia* Linn and *Nardostachys jatamansi* against experimental hepatotoxicity. Polyherbal suspensions were formulated using extracts showing significant activity and evaluated for both physicochemical and hepatoprotective activity in comparison with LIV-52 as standard. Petroleum ether (60-80°), chloroform, benzene, ethanol and aqueous extracts of *Ferula asafetida*, *Momordica charantia* Linn and *Nardostachys jatamansi* were evaluated for hepatoprotective activity against carbon tetrachloride-induced liver toxicity in Wistar rats. Polyherbal suspensions were prepared by the trituration method using a suspending agent and other excipients. Formulation F3 has shown significant hepatoprotective effect by reducing the elevated serum enzyme levels such as glutamate oxaloacetate transaminase, glutamate pyruvate transaminase and alkaline phosphatase. These biochemical observations were supplemented by histopathological examination of liver sections. Various parameters evaluated for all formulations were within the official specifications. Experimental data suggested that treatment with formulation F3 enhances the recovery from carbon tetra chloride-induced hepatotoxicity. From these results it may be concluded that the F3 formulation (containing chloroform, petroleum ether and aqueous extracts of *Ferula asafetida*, petroleum ether and ethanol extracts of *Momordica charantia* Linn. and petroleum ether and ethanol extracts of *Nardostachys jatamansi*) demonstrated significant hepatoprotective activity, that might be due to combined effect of all these extracts.

Recently there is a greater global interest in non synthetic, natural drugs derived from plant/herbal sources due to better tolerance and minimum adverse drug reactions^1^. No effective measures are available for the treatment of liver diseases in modern medicine so far. Herbal drugs, used in Indian systems of medicine are however claimed to be effective and safe in such ailments. These drugs are considered benign and are of particular value in the treatment of chronic diseases requiring prolonged therapy. Plant medicines are more often used in combination rather than in a single in order to get maximum benefit from their combined strength 2.

*Ferula asafoetida, Momordica charantia* and *Nardostachys jatamansi* are the medicinal plants used for centuries in the Ayurvedic system of medicine 3. These drugs are used for the treatment of various diseases such as abdominal pain, constipation, diarrhea, diabetes, epilepsy, hysteria and mental weakness 4–6. Thus the present study was undertaken to explore the effects of petroleum either, chloroform, benzene, ethanol and aqueous extracts of *Ferula asafoetida, Momordica charantia* and *Nardostachys jatamansi* against experimental hepatotoxicity and also to develop and evaluate polyherbal suspension formulations of the above extracts showing significant activity and to compare these effects with LIV-52 as standard marketed product.

Fruits of *Momordica charantia*, oleo gum resin of *Ferula asafoetida* and rhizomes of *Nardostachys jatamansi* were collected from the Pragati Ayurvedic Drug store, Belgaum and they were authenticated at Foundation for Revitalisation of Local Health Traditions (FRLHT), Bangalore. All the chemicals and solvents used were of either analytical or laboratory grade. Wistar rats were procured from Venkateshwara Enterprises, Bangalore. The animals were maintained at normal laboratory conditions and were given commercial laboratory animal feed and water *ad libitum*. The study was approved by the Institutional Animal Ethics Committee (Resl. No. 34/2005 CPCSEA)

Acute Toxicity studies 7 were carried out as per up and down methods according to OECD revised draft guidelines 423 October 2000. Based on these studies the doses were selected for the evaluation of hepatoprotective activity and development of suspension formulations. Petroleum ether, chloroform, benzene, ethanol and aqueous extracts of *Ferula asafoetida, Momordica Charantia* and *Nardostachys jatamansi* were obtained by maceration method 8 and they were screened for hepatoprotective activity against carbon tetrachloride-induced liver toxicity in Wistar rats. Based on these studies seven extracts were selected to formulate the suspensions.

Oral suspensions containing extracts showing significant hepatoprotective activity were prepared by trituration method using a suitable suspending agent and other excipients 9. Formulation F1 was prepared by using extracts showing maximum hepatoprotective activity. Formulation F2 was a combination of extracts showing moderate hepatoprotective activity. Formulation F3 was a combination of extracts of F1 and F2 both; Table 1 shows the formulation details. The prepared suspension formulations were evaluated for organolepetic characters such as colour, odor and taste 10. Physicochemical properties like sedimentation volume, pH, particle size, viscosity, zeta potential and redispersibility. Particle size analysis is done by optical microscopy. Viscosity of suspensions was determined by Brookfield viscometer type III, using spindle # 2 at 250 rpm. Zeta potential of suspensions was measured using Zeta meter 3±M 11. Redispersibility is measured by placing suspensions in a 100 ml graduated cylinder. After storage and sedimentation, the cylinder was rotated through 360° at 20 rpm. The end point is taken when the base of the cylinder is clear of sediment including the uniformity of suspended particles.

**TABLE 1:** FORMULATION COMPOSITION OF POLYHERBAL SUSPENSION

Ingredients (% w/w)	F1	F2	F3
*M. charantia* Pet ether extract	0.6667 g	--	0.285 g
*F. asafoetida* chloroform extract	1.6667 g	--	0.71428 g
*N. jatamansi* Pet ether extract	1.6667 g	--	0.71428 g
*M. charantia* alcohol extract	--	0.625 g	0.35714 g
*F. asafoetida* Pet ether extract	--	1.25 g	0.71428 g
*N. jatamansi* alcohol extract	--	1.25 g	0.71428 g
*F. asafoetida* water extract	--	1.25 g	0.71428 g
Tween 80	0.1%	0.1%	0.1%
Sodium CMC	2 g	2 g	2 g
Sucrose	10 g	10 g	10 g
Sorbitol	5 g	5 g	5 g
Methyl parabeen	0.20%	0.20%	0.20%
Lemon oil	0.01%	0.01%	0.01%
Purified water q. s.	100 ml	100 ml	100 ml

Quantities in grams per 100 ml suspension

Hepatoprotective activities of polyherbal formulations 12 were evaluated by carbon tetra chloride (CCl_4_)-induced hepatotoxicity method using Wister rats of either sex. For this rats were divided into six groups of six animals each. Group I was the control and rats received 1% Tween 80 in distilled water as vehicle p.o. Group II animals received CCl_4_ injections 0.7 ml/kg i.p. Group III rats were treated with 1 ml/kg Liv-52 orally. Group IV rats were treated with F1 formulation at a dose of 10 ml/kg orally. Group V received 10 mg/kg of F2 formulation orally and the Group VI received 10 mg/kg of F3 formulation orally. The above doses were given for ten successive days. CCl_4_-induced liver damage was produced by injecting 0.7 ml/kg of CCl_4_ intraperitonially on 3^rd^, 6^th^ and 10^th^ d to all the animals except group I. On the 10^th^ d, one hour after the last dose of CCl_4_ injection, the rats were sacrificed by cervical dislocation, blood was collected from carotid artery and serum was separated by centrifugation and used for evaluation of biochemical parameters such as glutamate oxaloacetate transaminase (SGOT), glutamate pyruvate transaminase (SGPT) 13 and alkaline phosphates (SALP) 14. Then the livers were carefully isolated and cleaned off extraneous tissue and preserved in 10 % neutral formalin solution and were subjected to histopathological studies.

Results obtained in the animal experiment were subjected for statistical analysis. The mean values were calculated as ±SE for each biochemical parameter. For determining the significant inter group difference each parameter was analyzed by one way ANOVA at *P* < 0.01% level of significance followed by Dunnet's test 15. Prepared formulations were evaluated for physicochemical stability such as sedimentation, viscosity, zeta-potential and pH by exposing the suspensions at 25° and 40° for a time period up to 30 d for all formulations 16.

Suspension formulations were prepared using combination of various extracts of *Ferula asafetida, Momordica charantia* and *Nardostachys jatamansi* that showed significant hepatoprotective activity. The suspensions were evaluated for the organolepetic, physicochemical and hepatoprotective activity. Table 2 shows the details of physicochemical properties of the suspensions and Table 3 shows the effects of suspension formulations on various biochemical parameters such as SGOT, SGPT, SALP and liver histopathological studies were carried out. In the organolepetic studies all polyherbal formulations exhibited pleasant appearance and acceptable odor. The average particle size of polyherbal suspension was in the range of 16.84 to 18.16 μm, the maximum viscosity was observed in F3 formulation (i.e. 53.8 cps), highest sedimentation volume was shown by formulation F1, pH of the formulations were found between 6.13 and 6.51, Zeta potential values were in the order of F2>F3>F1. Most of the formulation showed hepatoprotective activity and was significantly comparable with that of LIV-52. However the maximum hepatoprotective activity was found with formulation F3. The hepatoprotective activity was in the order of F3>F2>F1. There were no noticeable changes in sedimentation, viscosity and other physicochemical parameters after performing stability studies at variable temperature indicating formulations are stable and acceptable. There was no significant change in the physicochemical and organoleptic properties.

**TABLE 2:** PHYSICOCHEMICAL PARAMETERS OF FORMULATED SUSPENSIONS

Formulation	Sedimentation volume	Particle size (μm)	Viscosity (Cps)	pH	Redispersibility	Zeta potential (mV)	Density (gm/ml)
F1	0.29	17.84	50.8	6.13	Good	-11.3 to -36.8	1.0343
F2	0.26	18.16	52.0	6.33	Good	-14.5 to -43.7	1.0452
F3	0.21	16.84	53.8	6.51	Good	-12.1 to -41.1	1.0775

All values are mean of triplicates

**TABLE 3:** COMPARATIVE EFFECT OF FORMULATIONS AND CONTROL GROUP ON CCL_4_-INDUCED HEPATOTOXICITY IN RATS

Group	SGOT (IU/L)	SGPT (IU/L)	SALP (K.A units)
Control	224.0±19.85	112.0±5.26	147.5±04.62
CCL_4_	957.8±37.47	921.8±37.77	785.0±27.14
Liv.52	433.5±10.74**	208.5±05.84**	170.8±05.60**
F1	620.0±18.02**	363.8±19.97**	342.5±03.88**
F2	638.0±15.53**	300.2±06.96**	192.3±04.18**
F3	580.7±07.19**	314.3±10.42**	172.3±02.40**

** *P* < 0.01 level of significance

From the above result it may be concluded that the F3 formulation possesses significant hepatoprotective activity that might be due to combined effect of all significant extracts. However, this claim demands further study on isolation of individual components and observing their effect in the protection of liver against hepatotoxins for *Ferula asafetida*, *Momordica Charantia* and *Nardostachys Jatamansi*. Also the study needs elaborate technique to develop other suitable formulations from these extracts.
